# Taurolidine Lock Solutions for the Prevention of Catheter-Related Bloodstream Infections: A Systematic Review and Meta-Analysis of Randomized Controlled Trials

**DOI:** 10.1371/journal.pone.0079417

**Published:** 2013-11-21

**Authors:** Yong Liu, An-Qiang Zhang, Lin Cao, Hong-Tao Xia, Jun-Jie Ma

**Affiliations:** 1 Intensive care unit, Suining Central Hospital, Chuanshan District, Suining, Sichuan, China; 2 State Key Laboratory of Trauma, Burns and Combined Injury, Institute of Surgery Research, Daping Hospital, Third Military Medical University, Yuzhong District, Chongqing, China; San Raffaele Scientific Institute, Italy

## Abstract

**Background:**

Catheter-related bloodstream infections (CRBSIs) are a significant cause of morbidity and mortality in critically ill patients, contributing to prolonged hospital stays and increased costs. Whether taurolidine lock solutions (TLS) are beneficial for the prevention of CRBSIs remains controversial. In this meta-analysis, we aim to assess the efficacy of TLS for preventing CRBSIs.

**Methods:**

We conducted a systematic search of PubMed, EMBASE, and the Cochrane Central Register of Controlled Trials. Eligible studies included randomized controlled trials that reported on the effects of TLS for preventing CRBSIs. The primary outcome in these studies was catheter-related bloodstream infections, with microbial distribution of CRBSI and catheter-associated thrombosis as secondary outcomes. Data were combined using random-effects models owing to significant clinical heterogeneity.

**Results:**

Six randomized controlled trials (RCTs) conducted from 2004 through 2013 involving 431 patients and 86,078 catheter-days were included in the review. TLS were significantly associated with a lower incidence of CRBSIs when compared to heparin lock solutions (Risk Ratio [RR], 0.34; 95% Confidence Interval [CI], 0.21–0.55). Use of TLS significantly decreased the incidence of CRBSIs from gram-negative (G−) bacteria (P = 0.004; RR, 0.27; CI, 0.11–0.65), and was associated with a non-significant decrease in gram-positive (G+) bacterial infections (P = 0.07; RR, 0.41; CI, 0.15–1.09). No significant association was observed with TLS and catheter-associated thrombosis (RR, 1.99; CI, 0.75–5.28).

**Conclusions:**

The use of TLS reduced the incidence of CRBSIs without obvious adverse effects or bacterial resistance. However, the susceptibility of G+ and G- bacteria to taurolidine and the risk for catheter-associated thrombosis of TLS are indeterminate due to limited data. The results should be treated with caution due to the limited sample sizes and methodological deficiencies of included studies. Therefore, additional well-designed and adequately powered RCTs are needed to confirm these findings.

## Introduction

Central venous catheters (CVCs) are essential devices in patients receiving parenteral nutrition, chemotherapy and hemodialysis, and are often necessary to treat critically ill patients hospitalized in Intensive Care Units. However, use of these devices can lead to catheter-related bloodstream infections (CRBSIs) that are associated with increased morbidity and mortality rates, as well as extra costs. The attributable mortality rates for each infection are estimated at 11–24.8% in Europe and 12–25% in the United States [1], with an added cost ranging from $5,670 to $17,691 dollars per episode [2].

In long-term access devices, most CRBSIs are caused by intraluminal contamination, in other words, contamination from the infusate or the catheter hub and lumen [3]. Antimicrobial lock solutions seem to be an effective measure to control intraluminal contamination, especially for patients with a high-risk for infection. Some antibiotic catheter lock solutions have demonstrated a reduction in the risk for CRBSIs compared to heparin lock solutions [4], [5], [6], [7], [8], [9], [10], [11], but they are not routinely recommended due to the risk for development of antibiotic resistance [3], [12]. As a result of the emergence of isolates with reduced susceptibility and *in vitro* resistance to antibiotics, the need for alternative therapies to target CRBSIs has become apparent. Taurolidine [bis-(1,1-dioxoperhydro-1,2,4-thiadiazinyl-4)-methane], a derivative of the amino acid taurine, is an antimicrobial agent showing a broad spectrum of antimicrobial activity against both bacteria and fungi [13]. It is believed that methylol derivatives interact with components of bacterial cell walls resulting in irreparable injury. Taurolidine also appears to reduce adherence of bacteria to human epithelial cells *in vitro* [14]. Bacterial resistance has not been reported, as tarolidine’s mode of action resembles a disinfectant rather than an antibiotic [15].

A review by Bradshaw et al. [9] reported on 11 studies published between 1993 and 2006 on the use of taurolidine lock solutions (TLS) for the prevention of CRBSIs, although they did not conduct a meta-analysis, and were limited by small sample sizes. Recently, several more rigorous studies evaluating the association between TLS and CRBSIs risk have been published [16], [17], [18], [19], [20], [21], [22], [23], [24], [25], [26]. We therefore performed a systematic review and meta-analysis of all randomized controlled trials (RCTs) to determine the efficacy of TLS for the prevention of CRBSIs.

## Methods

### Literature Search

We conducted a systematic literature search of PubMed, EMBASE and the Cochrane Central Register of Controlled Trials for all relevant articles. The search process was designed to find all initial trials involving terms: ‘taurolidine’, ‘taurolin’ ‘tauroflex’, ‘tauroline’, ‘catheter’, ‘catheterization’, ‘infection’, ‘bacteremia’ and ‘sepsis’. Other potentially relevant studies were sought in references of all selected studies, relevant conference proceedings, trial registries, and ongoing trial databases. When needed, study authors were contacted for further information. The search strategy was according to the Preferred Reporting Items for Systematic Reviews and Meta-analysis (PRISMA) statement [27]. Databases selected were searched in April 2013 and an automatic alert for new articles fitting the search criteria was included through July 2013. The detailed search strategies are presented in file S1.

### Selection Criteria

All RCTs comparing TLS (with or without anticoagulant) to heparin lock solutions for the prevention of CRBSIs were included. No restrictions regarding language, publication status, sample size or state were applied. Retrospective studies, non-randomized trials, case reports and single arm trials were excluded. Patients of all ages were eligible for the study if they needed an intravascular catheter for facilitating the administration of intravenous therapy, irrespective of size, type, and number of lumens. The major exclusion criteria included patients with active, recent infections or were under antibiotic therapy.

### Data Collection and Quality Assessment

Data were extracted independently by two reviewers (YL and AQZ) using a pre-designed data extraction form including data source, eligibility, methods, participant characteristics, interventions and results. The primary outcomes assessed were catheter-related bloodstream infections (all published CRBSI definitions were accepted). Secondary outcomes included distribution of bacteria (gram positive or gram negative) and catheter-associated thrombosis (defined as thrombosis or need for thrombolytic therapy or removal of the catheter because of flow problems), exit site infections, biofilm, emergence of resistance, and adverse events. Findings from the two reviewers were compared and compiled. Discrepancies were resolved by discussion, or by consultation with the 3rd author (LC) if needed. Missing data were requested from the original authors through electronic correspondence or mail. We described and assessed allocation concealment, sequence generation, blinding, incomplete outcome data and selective outcome reporting individually and according to the Cochrane Collaboration’s tool for assessing bias.

### Data Synthesis and Statistical Analysis

RevMan 5.2 was used to perform the meta-analysis [28]. Outcomes are presented as risk ratios (RR) for categorical data with 95% confidence intervals (CI). P-values less than 0.05 were considered statistically significant. Statistical heterogeneity among trials was evaluated using both the Cochrane’s Q and I^2^ statistics [29], with a value of 0% indicating no observed heterogeneity. I^2^ values of 25%, 50%, and 75% were described as low, moderate, and high, respectively, according to Higgins et al. [30]. Since there was significant clinical heterogeneity, we used the random-effects model to combine the effect sizes of the included studies. We did not assess publication bias due to the small number of included studies.

## Results

### Study Characteristics

**Figure 1:**
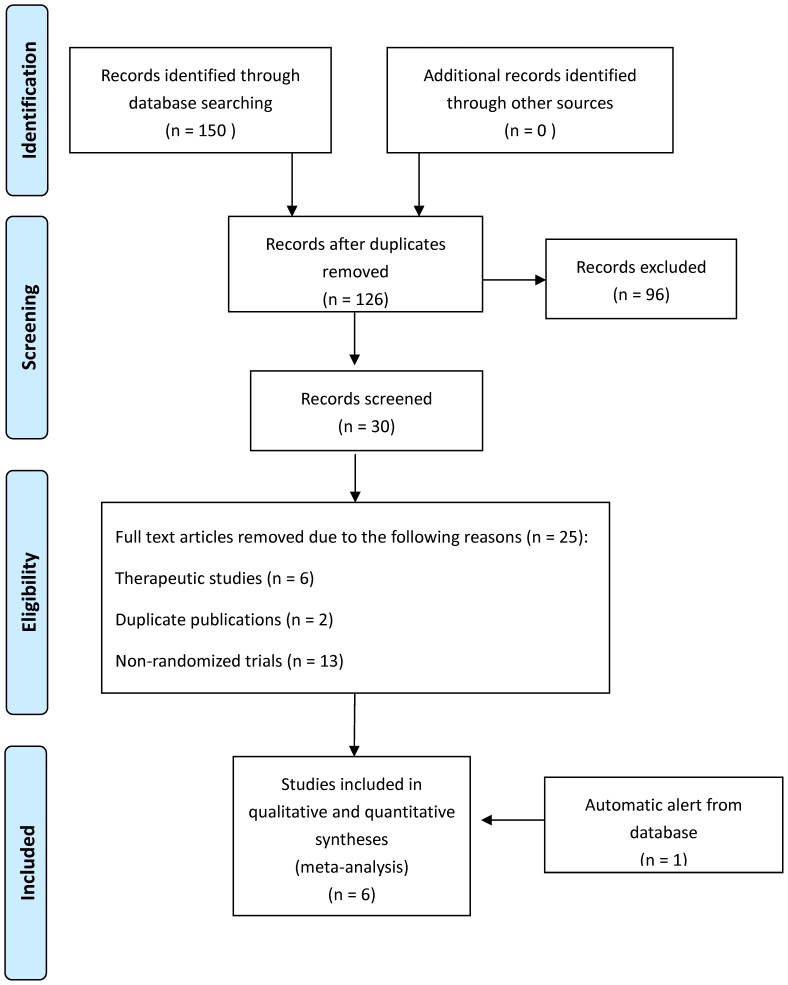
Flowchart for selection of trials.

The database search yielded 126 studies, of which 96 were irrelevant. Thirty studies were further evaluated, of which 25 were excluded (Figure 1) [16], [17], [20], [21], [23], [24], [31], [32], [33], [34], [35], [36], [37], [38], [39], [40], [41], [42], [43], [44], [45], [46], [47], [48], [49]. The last article fitting the search criteria was supplied by an automatic alert from Pubmed [26]. Altogether, six RCTs, conducted from 2004 through 2013, were included in the review [18], [19], [22], [25], [26], [50]. Table 1 summarizes the baseline characteristics of the included studies and their participants, including hemodialysis, parenteral nutrition and pediatric oncology patients. The number of patients included in every study ranged from 30 to 112, the age of participants in the trials ranged from 5 to 58, and the follow-up period for participants ranged from 31 to 349 days.

**Table 1:** Study characteristics.

Study	Participants	Interventions	Location	Studyyears	No. ofpatients	Follow-up(days)	Meanage(years)	Diabetes(%)	Type ofcatheters;T/NT/Port (%)	Definition of CRBSI	Fundingbypharma
Betjes2004	hemodialysispatients	1.35% T +4%C, H (5000 U/ml)	Netherlands	2002–2003	58	59	54	27.6	24/76/0	Symptom, culture,source	No
Bisseling2010	PediatricHPNpatients	2% T, H(150 U/ml, 5 ml)	Netherlands	2006–2008	30	344	52	NA	63/0/37	Symptom, culture,source, both positive	No
Solomon2010	hemodialysispatients	1.35% T and 4%C, H (5000 U/ml)	England	2006–2008	107	166	58	11.2	100/0/0	Culture	No
Dumichen2012	pediatriconcologypatients	1.35% T +4% C,H (100 U/ml)	Germany	2007–2008	71	194	8.2	NA	100/0/0	Symptom, culture,source	Tauropharm
Handrup2013	pediatriconcologypatients	1.35% T +4% C,H (100 IU/ml)	Denmark	2008–2010	112	349	Median 6 vs. 5	NA	12/0/86	Symptom, culture,source, Both positive,time difference	Tauropharm
Zwiech2013	hemodialysispatients	1.35% T+4% C,H (500 IU),H (5000 U/ml)	Poland	NA	53	31	57	53.3	58/42/0	NA	No

**NOTE.** T/NT/Port, Tunneled/Non-tunneled/Port catheters; HPN, home parenteral nutrition; RCT, randomized controlled trial; T, taurolidine; C, citrate; H, heparin; Symptom, clinical signs and symptoms; Culture, positive blood culture; Source, no other source of infection; Both positive, a positive blood culture from the central venous catheter and peripheral vein for the same organism; time difference, growth of microbes from a blood sample drawn from a catheter at least 2 h before blood sample of a peripheral vein; NA: not available.

### Risk of Bias Assessment Results

**Figure 2:**
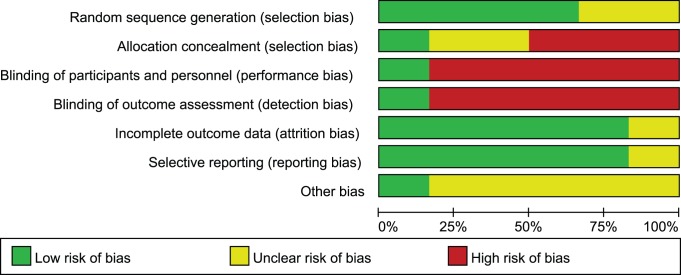
Risk of bias graph. Review authors’ judgements for each ‘Risk of bias’ item presented as percentages across all included studies.

Five of the six included trials did not provide sufficient details of an adequate method of allocation concealment and blinding to qualify them as possessing risk of selection and performance bias [18], [22], [25], [26], [50]. One of these trials was judged as unclear for risk of bias assessment as most of the domains of methodological quality were not reported [26] (Figure 2).

### Catheter-related Bloodstream Infections

**Figure 3:**
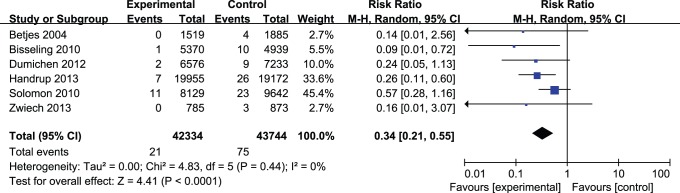
Forest plot for the incidence of CRBSIs per catheter-day between TLS and control groups.

Data for the effect of TLS (with or without anticoagulant) versus control (heparin) lock solutions for the prevention of CRBSIs were available from six trials [18], [19], [22], [25], [26], [50]. The overall pooled risk ratio (RR) of CRBSIs using a random effects model was 0.34 (CI, 0.21–0.55; P<0.0001) without significant heterogeneity (P = 0.44), indicating a significant trend towards benefit in patients who received TLS (Figure 3).

### Types of Microorganisms

**Figure 4:**
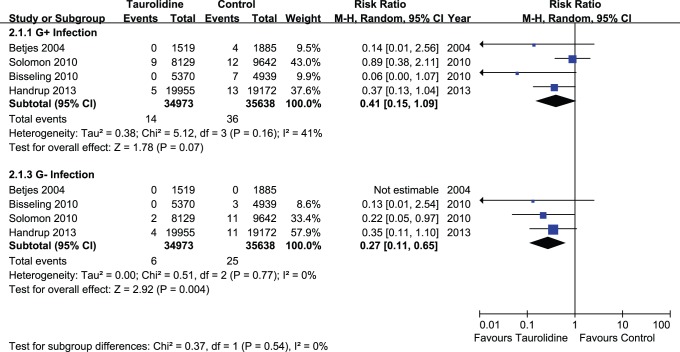
Microorganism distribution between G+ and G- infections.

Complete data on the type of microorganisms leading to CRBSIs were available from four trials [18], [19], [25], [50]. The number of CRBSIs by gram-negative (G−) bacteria was significantly decreased after TLS use (P = 0.004; RR, 0.27; CI, 0.11–0.65), whereas the decrease of gram-positive (G+) bacterial infection was not significant (P = 0.07; RR, 0.41; CI, 0.15–1.09), without significant heterogeneity (P>0.10) (Figure 4).

### Catheter-related Thrombotic Complications

**Figure 5:**
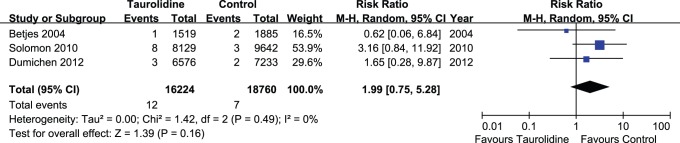
Forest plot for the incidence of catheter occlusion by thrombosis between TLS and control groups.

Data for catheter occlusion by thrombosis in TLS and control groups were available from three trials [19], [22], [50]. There were no significant differences between TLS-treated and control-treated groups for catheter occlusions by thrombosis (P = 0.16; RR, 1.99; CI, 0.75–5.28), without significant heterogeneity (P = 0.49) (Figure 5), though Solomon et al. reported that use of TLS was associated with a greater need for thrombolytic treatment [19].

### Exit Site Infections

**Figure 6:**
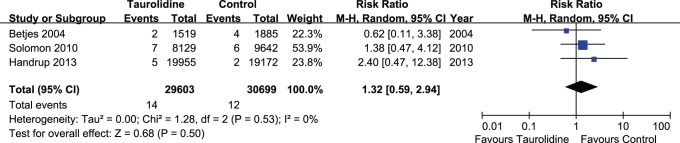
Forest plot for the incidence of exit site infections between TLS and control groups.

Data for the incidence of exit site infections in TLS and control groups were available from 3 trials [19], [25], [50]. There were no significant differences between TLS-treated and control-treated groups in the outcomes of exit site infections per catheter-day (RR, 1.32; CI, 0.59–2.94; P = 0.50) without significant heterogeneity (P = 0.53) (Figure 6).

### Biofilm and Colonization

The study by Dumichen et al. reported no significant difference in biofilm growth between groups, with 7 of 26 catheters in the control group and 6 of 25 in the TLS group colonized by bacteria at time of removal [22]. Additionally, a study by Handrup et al. using scanning electron microscopy also found no difference in the biofilm development between TLS and control groups, as positive cultures were found from samples of all six of the catheters examined [36].

### Adverse-effects and Bacterial Resistance

Four of the six included trials did not report any adverse-effects associated with TLS treatment. However, Dumichen et al. reported that 20% of TLS-treated patients experienced adverse-effects (nausea, vomiting, abnormal taste sensations) [22]. Handrup et al. documented that some patients in their TLS-treated group reported a brief, unpleasant taste when their CVCs were flushed with the taurolidine solution [25]. There were no reports of bacterial resistance to taurolidine in any of the included studies.

## Discussion

In this meta-analysis, we found that TLS-treatment was associated with a reduced CRBSI rate compared to treatment with control lock solutions. We also found that the CRBSI rate was significantly decreased in some non-randomized trials concerning high-risk patients [21], [24], [48], [49]. However, Filiopoulos et al. found no statistically significant difference in the incidence of CRBSIs between TLS and control groups [20]. Taken together, these currently available data suggest that TLS treatment is as effective as, if not better than, treatment with antibiotics for the prevention of CRBSIs. Further studies are needed to enhance the quality of experimental design and evaluate development of microbial resistance and catheter-related thrombosis as a result of TLS treatment, as well as examine in more detail which patients would benefit most from TLS treatment.

The Healthcare Infection Control Practices Advisory Committee guidelines (HICPAC) currently recommend antibiotic lock solutions only as a Class II recommendation in those who have long-term catheters and a history of multiple CRBSIs [51]. In our review, only one study recruited patients with high-risk of infection [18]. This standard was implemented by HICPAC to prevent the generation of resistant bacteria. However, there have been no reports of bacterial resistance with TLS treatment, to our knowledge. Although taurolidine demonstrates antimicrobial properties, it is a biocide rather than an antibiotic [14]. Antibiotics interact specifically with structures or metabolic processes of the microorganisms, while biocides inactivate the microorganism through rather unspecific or multifactorial ways. According to Olthof et al., long-term use with increasing concentrations of taurolidine did not lead to selective growth of microorganisms [52]. Exposure to sub-lethal concentrations of the biocide has been reported to result in decreased antimicrobial activity. However, this adaptation is reversible, and sub-culturing the bacteria without the respective substance restores sensitivity to the agent [53]. While most studies show that clinical isolates are susceptible to correctly applied biocides, further studies are needed to understand the mechanisms of antimicrobial resistance.

According to the study by Olthof et al. [52], a 50% minimum inhibitory concentration (MIC50) for the majority of common G+ or G- bacteria was less than 512 mg/l (2048 mg/l for *Candida albicans*). The concentration of taurolidine typically used for lock solutions (1.35%–2%) is at least 10 times higher than the MIC50. However, the frequency of TLS utilization was varied among included studies. The CVCs in most of the studies were locked after each treatment cycle or hemodialysis, while some catheters were locked once a week or a once a month (later for completely implantable devices) [25]. No studies reported on the degree of decrease in taurolidine concentrations in the catheter lumen, or on the relationship between the decline of concentration and CRBSIs.

There were no significant differences between G+ and G- bacteria in taurolidine minimum inhibitory concentrations reported in the study by Olthof et al. [52]. However, the antimicrobial effect of taurolidine against G+ and G- bacteria was inconsistent among studies. Filiopoulos et al. and Solomon et al. reported that G- infections were depressed more significantly than G+ infections after use of TLS [19], [20]. Conversely, Simon et al. found a more robust decrease in G+ infections [16]. These differences could be attributed to the relatively small sample sizes in these studies and the variation of microbial florae among hospitals.

A total of 19 catheter occlusions caused by thrombopoiesis were reported in the included studies, and meta-analysis revealed no significant difference between TLS and control groups. However, Solomon et al. [19] reported an increased risk of thrombosis in their TLS group compared with the control heparin group. For this reason, they conducted thrombolytic therapy with a tissue plasminogen activator or urokinase. Furthermore, some clinicians added heparin to TLS to reduce the risk of thrombosis [17], [23]. A later study by Solomon et al. [23] in 2012 found that taurolidine-citrate-heparin lock solutions reduced the need for thrombolysis compared to taurolidine-citrate alone. The lack of a significant association between TLS and catheter-associated thrombosis in our review may be a result of insufficient sample sizes, therefore additional studies are needed to resolve this discrepancy.

Two studies included in our analyses reported temporarily minor adverse reactions in pediatric oncology patients after receiving TLS, including nausea, vomiting, abnormal taste sensations and tingling sensations. However, only four patients withdrew from trials because of these adverse effects.

Several limitations of our conclusions are listed below. Firstly, only one [19] of the six included studies was a rigorously conducted RCT, and the study by Zwiech et al. [26] had serious methodological deficiencies. Secondly, antimicrobial lock solutions are suitable for patients with high-risk of infection according to the HICPAC standard; yet only one study in our review recruited such patients. Thirdly, two studies used antibiotics to prevent infection. One trial used mupirocin nasal decontamination [50] and another trial used cotrimoxazole for the prevention of *Pneumocystis jiroveci* pneumonia [22], which might affect the performance of TLS and result in false conclusions. Finally, there are various other factors that may affect the accuracy of our results, such as differences in patient types, catheter types, sample sizes, follow-up durations, definition of CRBSI, etc. Therefore, the results of this meta-analysis should be considered with caution. Additional high quality large-scale clinical trials with adequate statistical power are needed to further evaluate the effect of TLS on the prevention of CRBSIs before we can come to a definite conclusion.

## Conclusions

The results of our analyses suggest that TLS reduced the incidence of CRBSIs without obvious adverse effects and bacterial resistance. There was insufficient evidence to demonstrate a difference in susceptibility to taurolidine between G+ and G- bacteria. As the analyses were limited to studies with small sample sizes, we cannot conclude whether TLS treatment is associated with a higher risk for catheter-associated thrombosis compared to a control heparin lock solution. In addition, we must also treat the results with caution due to methodological deficiencies of the included studies. More well-designed and adequately powered RCTs are needed to confirm these findings.

## Supporting Information

File S1 **Electronic search strategies for Pubmed, Central, and EMBASE.**
(RAR)
